# Diffuse Reflectance Spectroscopy with Dilution: A Powerful Method for Halide Perovskites Study

**DOI:** 10.3390/molecules28010350

**Published:** 2023-01-01

**Authors:** Aleksei O. Murzin, Anna Yu. Samsonova, Constantinos C. Stoumpos, Nikita I. Selivanov, Alexei V. Emeline, Yury V. Kapitonov

**Affiliations:** 1 Photonics of Crystals Laboratory, Saint Petersburg State University, Ulyanovskaya d.1, St. Petersburg 198504, Russia; 2 Department of Materials Science and Technology, University of Crete, Voutes, GR-70013 Heraklion, Greece

**Keywords:** halide perovskites, diffuse reflectance spectroscopy, absorption, excitons

## Abstract

Halide perovskites and their low-dimensional analogs are promising semiconductor materials for solar cells, LEDs, lasers, detectors and other applications in the area of photonics. The most informative optical property of semiconductor photonics materials is the absorption spectrum enabling observation of the fundamental absorption edge, exciton structure, defect-related bands, etc. Traditionally, in the study of halide perovskites, this spectrum is obtained by absorption spectroscopy of thin films or diffuse reflectance spectroscopy of powders. The first method is applicable only to compounds with the developed thin film deposition technology, and in the second case, a large absorption coefficient narrows the observations down to the sample transparency region. In this paper, we suggest the diffuse reflectance spectroscopy with dilution as a method for obtaining the full-range absorption spectrum from halide perovskite powders, and demonstrate its application to practically important cases.

## 1. Introduction

Halide perovskites burst into the world of photonics and optoelectronics as a material for solar cell absorbers [1], but soon showed their applicability for lasers [2], LEDs [3], X-ray [4] and photodetectors [5]. For halide perovskites, as for all other semiconductors, determinative parameters are the band gap, exciton binding energy and oscillator strengths of transitions. One needs also to evaluate the role of defects in electronic structure and quantum-size effect in low-dimensional perovskite-like compounds. All of these material characteristics can be traced back to the electronic structure and could be studied experimentally using various optical methods, the basic ones of which are photoluminescence (PL) and absorption spectroscopy.

PL spectroscopy is based on the optical excitation of the sample above the band gap and detection of the emitted light. The requirement for this method is the presence of emission, which is not met in many halide perovskites at room temperature, in the presence of non-radiative recombination centers or for indirect band gap materials. PL gives information about lowest lying states in the sample which are not necessary the states with high oscillator strength, but could be doping-related or defect states [6], or self-trapped excitons (STE) [7]. In the presence of emission the PL excitation (PLE) spectrum can be measured by scanning the excitation light energy. Taking into account all the above complexities the PLE spectrum could be taken as the estimation of the absorption spectrum.

Absorption spectroscopy gives information about optical transitions with corresponding oscillator strengths and could be used also for non-emitting samples. The most straightforward method for absorption determination is the measurement of the spectrum of the light transmitted through a slab of the material. For semiconductor materials the absorption coefficient in the fundamental absorption range is $\alpha >10^{4}$ cm^−1^ so the slab should have sub-micrometer thickness in order to light to pass through. Although many of halide perovskite materials are grown as single crystals, fabrication of thin slabs from them is limited by the brittleness of the material [8] with only exception for exfoliation techniques of 2D haloplumbates with low interlayer connectivity [9,10]. For this reason, polycrystalline thin films are the common samples for transmittance measurements. Fabrication of such films is a separate technological issue solved only for limited number of halide perovskite materials, such as MAPbI_3_ (MA^+^ = CH_3_${\mathrm{NH}}_{3}^{+}$) and few others [11].

For the rest of halide perovskites the method of choice for absorption measurements is the diffuse reflectance spectroscopy (DRS) of powders. Typically this method is implemented in a spectrophotometer equipped with an integrating sphere. First the signal from the known non-absorbing (”white”) reference material is collected. Then the holder filled with the grained sample is placed instead of the reference. The reflectivity signal *R* is measured. The simplest way to visualize the portion of light not leaving the sample is to plot the function A=1−R. In the assumption of the infinitely thick sample the Kubelka–Munk transformation [12,13,14] could be used to determine pseudo-absorbance (remission):

(1) $$F=\frac{{\left(1-R\right)}^{2}}{2R}=\frac{K}{S},$$

where *K* is absorption and *S* is scattering coefficients of the studied material. Although the F(E) function is usually taken as a close approximation of the absorption spectrum, care should be taken for strongly absorbing materials. The almost absent reflectivity from the sample (R→0) leads to the undefined value of *F* being very sensitive to any experimental errors. DRS measurements of semiconductors without the sample dilution allows to reliably determine only the defect-related absorption and the long-wavelength tail of the fundamental absorption. Thus, despite the general prevalence of the DRS equipment in labs, in the study of halide perovskites, with rare exceptions, DRS is used for obtaining nonsupporting data, and sometimes even for drawing incorrect conclusions. Also, we would like to point out that in halide perovskites it is necessary to use the Tauc formalism [15,16,17] with great care, since it is applicable for interband transitions in the absence of any absorption below the bandgap $E_{g}$ [18]. The presence of strong exciton transitions below $E_{g}$ requires the use instead the Elliot formalism [19,20] that takes such transitions into account. The presence of exciton transitions can also lead to the misinterpretation of them as the Urbach tail.

With this work, we would like to show that the well-known method of DRS with dilution allows obtaining reliable data in a variety of practical-important cases of halide perovskites study.

## 2. Results and Discussion

For DRS measurements with dilution the sample powder is diluted by a powder of transparent (”white”) matrix. For the visible range measurements the BaSO_4_ is the material of choice due to its nonreactivity and transparency at E<3.5 eV [21]. Let us denote the volume fraction β in the following way:

(2) $$\beta =\frac{V_{1}}{V_{1}+V_{2}},$$

where $V_{1,2}$ are volumes of the sample and matrix respectively. The condition of transparency of the matrix material means that its absorption coefficient $K_{2}=0$ in the region of interest. In this case the absorption and transmission coefficients of the mixture will be equal to $K=\beta K_{1}$ and $S=\beta S_{1}+\left(1-\beta \right)S_{2}$, respectively. Here we designated the absorption and scattering coefficients of the sample as $K_{1}$ and $S_{1}$, and the scattering coefficient of the matrix as $S_{2}$. Substitution of these expressions in Equation (1) leads to the following equation for pseudo-absorbance of the mixture:

(3) $$F\left(\beta \right)=\frac{K_{1}}{S_{2}}\cdot \frac{\beta }{1-\left(1-\frac{S_{1}}{S_{2}}\right)\beta }.$$

This equation could be used to fit experimental F(β) series and to independently determine the $\frac{K_{1}}{S_{2}}$ and $\frac{S_{1}}{S_{2}}$ values. Usually the scattering coefficient of the matrix in the transparency energy range is monotonic and slowly changing, so the functional behaviour of $K_{1}$ and $S_{1}$ could be determined. We would like to emphasise that such approach could be used to untangle absorption and scattering of studied material opposed to Kubelka–Munk transformation of single DRS spectrum. The minimal series of dilutions consist of only two DRS spectra with different fractions. In this case Equation (3) leads to a simple set of equations that could be solved for $\frac{K_{1}}{S_{2}}$ and $\frac{S_{1}}{S_{2}}$ (see Methods). The choice of an undiluted sample (β=1) as one of the points simplifies the experiment. As the second point we recommend choosing a fraction with β<0.01. When preparing a mixture of materials by mass, a formula should be used that takes into account materials densities (see Methods).

**Figure 1:**
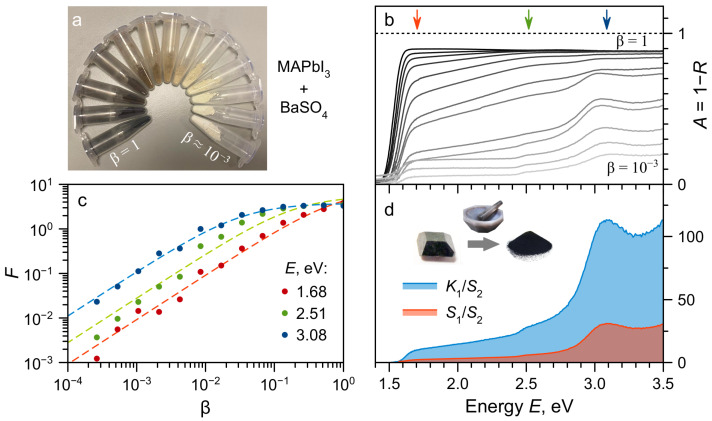
(**a**) Vials with mixtures of MAPbI_3_ and BaSO_4_ powders starting from undiluted MAPbI_3_ (β=1) and halving the mass fraction for every next vial clockwise. Note the appearance of the material colour at β>0.1 (sixth and further vials). (**b**) DRS spectra of powders mixtures from (**a**). (**c**) Dependence of the Kubelka–Munk transformation *F* of DRS data on the volume fraction β for different light energies (dots). Dots color corresponds to the energies marked by arows in (**b**). Dashed curves—fit by Equation (3). (**d**) Relative absorption $\frac{K_{1}}{S_{2}}$ and scattering $\frac{S_{1}}{S_{2}}$ coefficients of MAPbI_3_ extracted from DRS data fitting. Inset shows the pulverization process schematically.

For a detailed illustration of the method, we chose the most studied and practically demanded three-dimensional halide perovskite MAPbI_3_ (MA^+^ = CH_3_${\mathrm{NH}}_{3}^{+}$). Single crystals of MAPbI_3_ were synthesised using the counterdiffusion-in-gel method [22]. The crystals were pulverized and mixed with BaSO_4_ powder. Figure 1a shows vials with mixed powders starting from pure MAPbI_3_ (β=1) with each following is diluted twice by mass with BaSO_4_. The DRS data in the form of A=1−R are shown in Figure 1b. Note the ”saturation” of the spectra for β→1 due to the total absorption of light in the sample. Using these data to analyze the energy structure of the sample can lead to incorrect conclusions. For example, the measurement of the band gap energy using the apparent absorption edge could lead to the band gap underestimation. These data also do not allow extracting information about the energy structure of transitions in the absorption region. To obtain complete information about the spectral properties of the sample, the entire data array should be analyzed. Figure 1c shows the dependencies of the Kubelka–Munk transformation of DRS data on the volume fraction for three different energies (marked by arrows in Figure 1b). These sections could be fitted by Equation (3) yielding the relative absorption and scattering coefficients. Same fitting procedure was used for the whole set of DRS data. Figure 1d shows the relative absorption and scattering spectra for MAPbI_3_ material. The fundamental absorption of the material began around 1.6 eV, with two absorption edges of higher-lying states could be observed around 2.5 and 3.0 eV. This data is in a good agreement with transmission measurements of MAPbI_3_ thin films [23,24]. In this work, we do not want to discuss the nature of the observed states any further. However we would like to note that a correct description of interband absorption in a direct-gap semiconductor even above the exciton dissociation temperature requires the use of the Elliot formalism, which takes into account excitonic, as well as the electron-hole correlation effects. The relative absorption and relative scattering (Figure 1d) show similar spectral behaviour. Thus, their ratio in the expression for the Kubelka–Munk transformation (Equation (1)) is not very informative. One of the advantages of the proposed method of DRS with dilution is the ability to separate these contributions.

The proposed method allows obtaining reliable spectroscopic data for other cases of practical importance for the study of halide perovskites. A hot area of research is the study of low-dimensional perovskite-like compounds. Quantum-size effects in these materials lead to a significant blue shift in the band gap of the material and an increase in the exciton binding energy to hundreds of meV [25,26]. The stability of a free exciton at room temperature and the complex emission routes involving self-trapped excitons [7] make measurements of the absorption of these materials both important and difficult task. To demonstrate the applicability of the proposed method to low-dimensional compounds, we chose the PyPbI_3_ (Py^+^ = C_5_H_5_NH) single crystal [27]. The crystal lattice of this material is the package of one-dimensional inorganic chains separated by an organic pyridinium cations. Restriction of electron and hole movement in the inorganic chain makes the material a natural quantum wire structure with band gap blue-shifted to near-UV region in comparison to the near-IR band gap of 3D iodide perovskites.

**Figure 2:**
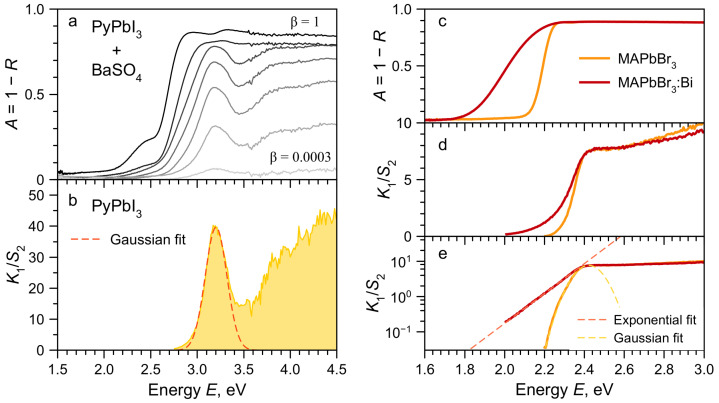
(**a**) DRS spectra of powders mixtures of PyPbI_3_ and BaSO_4_ for different mass fractions β. (**b**) Relative absorption $\frac{K_{1}}{S_{2}}$ coefficients of PyPbI_3_ extracted from DRS data fitting. Dashed line—Gaussian fit of the excitonic resonance. (**c**) DRS spectra and relative absorption $\frac{K_{1}}{S_{2}}$ coefficients in linear (**d**) and log (**e**) scales for undiluted MAPbBr_3_ (orange curves) and MAPbBr_3_:Bi (red curves) powders. Dashed curves in (**e**) are fits by Gaussian (orange) and exponent (red) functions.

As earlier, PyPbI_3_ single crystals grown by counterdiffusion-in-gel method were pulverized and mixed with BaSO_4_ powder with different volume fractions β. Figure 2a shows the DRS spectra of mixtures. The DRS from undiluted sample (β=1) shows the total absorption above 2.9 eV with features corresponding to experimental imperfections rather than the actual material resonances. The only reliably recorded state is the weak absorption band around 2.4 eV in the transparency region of the sample. Much more informative is the analysis of the full set of DRS data using proposed method. Figure 2b shows the obtained relative absorption spectrum of PyPbI_3_. The spectra is dominated by the free exciton absorption peak centred at 3.20 eV. This peak has Gaussian lineshape with FWHM = 0.27 eV indicating the inhomogeneous broadening of the transition. The exciton absorption peak is followed by the interband absorption. The gap between the excitonic and interband (electron-hole) absorption indicates the high exciton binding energy on the order of 0.5 eV which is typical for such low-dimensional structures. Obtaining such information for PyPbI_3_ using other methods is difficult, since there is no developed technology for deposition of thin films this material, and PLE measurements are impossible, since the material does not show any luminescence at room temperature.

Doping is an important area of research for halide perovskites. The introduction of impurities, as well as the presence of intrinsic defects in crystals, can introduce significant changes in their absorption spectra in the form of absorption bands in the transparency region of the material or tail states. On the other hand, the doping can also lead to a change in the fundamental absorption, for example, to the band gap shift. The described method allows one to obtain accurate data on the absorption spectra in this case as well. To demonstrate this, we took the example of bismuth doping of MAPbBr_3_ halide perovskite [28]. Two single crystals were synthesised, pulverized and studied: nominally pure MAPbBr_3_ and MAPbBr_3_ doped with Bi^3+^. Figure 2c shows the DRS spectra for the undiluted sample powders. The only conclusion that can be drawn from these data is that states appear in the MAPbBr_3_ transparency region under doping. At the same time, it is not possible to establish the nature of these states. Figure 2d,e shows the relative absorption spectra of both materials obtained by the DRS with dilution method. In both cases, the spectral position of the interband absorption edge coincides, which indicates the invariance of the band gap upon doping [28]. The long-wavelength absorption edge in the case of an undoped material is well described by the Gaussian, which is a manifestation of its excitonic nature. The absorption of defect states arising upon doping with bismuth is well described by an exponential dependence, which is a typical manifestation of the so-called Urbach tail.

## 3. Materials and Methods

### 3.1. Reagents and Crystal Growth

Lead(II) bromide PbBr_2_ (98%, Sigma-Aldrich, St. Louis, MO, USA), lead(II) iodide PbI_2_ (99%, Sigma-Aldrich), hydrobromic acid HBr (40% in H_2_O, Iodobrom, Crimea), hydroiodic acid HI (56% in H_2_O, Iodobrom), hypophosphorous acid H_3_PO_2_ (50% in H_2_O, Acros Organics, Geel, Belgium), methylamine CH_3_NH_2_ (38% in H_2_O, Lenreactiv, Saint Petersburg, Russia), pyridine (99.8% Sigma-Aldrich) and BiBr_3_ (98% Sigma-Aldrich) were used as received. Silica gel was prepared from sodium metasilicatecrystallohydrate solution Na_2_SiO_3_·9H_2_O with the distilled water as solvent. To stabilize the hydroiodic acid, hypophosphorous acid was added to it in the 9:1 volume ratio. All solutions and sols, where HI was used as a solvent or reagent, were prepared using this stabilized solution with H_3_PO_2_.

The counterdiffusion-in-gel growth method [22] developed by us was used for crystal growth of single crystals of materials studied. The glass U-tubes were filled with silica gel and solutions of lead halides and amines (methylamine, pyridine) in hydrohalic acid with 1 M concentrations. For Bi^3+^-doped crystals the BiBr_3_ was used with 1 M concentration in the solution.

### 3.2. Basic Characterization

In order to prove the single phase nature of synthesised materials the X-ray diffraction was recorded from crushed crystals with a high resolution X-ray diffractometer Bruker D8 Discover using a long focus X-ray tube CuKα anode. Reflected X-rays were detected using a solid position-sensitive detector LYNXEYE. Measurements were carried out at room temperature and proved the known crystal structure and single-phase nature of studied samples.

### 3.3. DRS Measurements

The grown single crystals were crushed in an agate mortar. Next, a series of diluted mixtures was prepared: at each next stage, the previous powder was diluted twice by weight with BaSO_4_ powder. The volume fraction was determined from known material densities: ρ(BaSO_4_) = 4.5 g/cm^3^, ρ(MAPbI_3_) = 4.159 g/cm^3^ [29], ρ(MAPbBr_3_) = 3.83 g/cm^3^ [30], ρ(PyPbI_3_) = 3.837 g/cm^3^ [27]. The DRS spectra were recorded by the Cary 5000 UV-Vis-NIR spectrometer equipped with a diffuse reflectance apparatus.

### 3.4. DRS Spectra Fitting for Two Dilutions

Let the $F_{1}$ and $F_{2}$ values are obtained when measuring DRS for two fractions $\beta_{1}$ and $\beta_{2}$. In this case material parameters could be found in the following way:

(4) $$\left\{\begin{array}{c} \frac{K_{1}}{S_{2}}=\frac{F_{1}F_{2}}{F_{1}-F_{2}}\cdot \frac{\beta_{1}\beta_{2}}{\beta_{1}-\beta_{2}},\\ \frac{S_{1}}{S_{2}}=\frac{F_{1}F_{2}}{F_{1}-F_{2}}\left(\frac{1-\beta_{1}}{F_{1}\beta_{1}}-\frac{1-\beta_{2}}{F_{2}\beta_{2}}\right). \end{array}\right.$$

The expression further simplifies if the undiluted sample is taken as the first point ($\beta_{1}=1$):

(5) $$\left\{\begin{array}{c} \frac{K_{1}}{S_{2}}=\frac{F_{1}F_{2}}{F_{1}-F_{2}}\cdot \frac{\beta_{2}}{1-\beta_{2}},\\ \frac{S_{1}}{S_{2}}=\frac{F_{1}}{F_{2}-F_{1}}\cdot \frac{1-\beta_{2}}{\beta_{2}}. \end{array}\right.$$

### 3.5. Conversion between Mass and Volume Fractions

Let us denote the mass fraction as $\gamma =\frac{m_{1}}{m_{1}+m_{2}}$, where $m_{1,2}$ are masses of the sample and the matrix powders. Volumes of materials could be determined as $V_{1,2}=\frac{m_{1,2}}{\rho_{1,2}}$, where $\rho_{1,2}$ are materials densities. The volume fraction could be found as:

(6) $$\beta =\frac{\rho_{2}}{\rho_{1}}\cdot \frac{\gamma }{1-\gamma \left(1-\frac{\rho_{2}}{\rho_{1}}\right)}.$$

Substitution of β into Equation (3) leads to the following equation for F(γ) value:

(7) $$F\left(\gamma \right)=\frac{K_{1}}{S_{2}}\cdot \frac{\rho_{2}}{\rho_{1}}\cdot \frac{\gamma }{1-\left(1-\frac{S_{1}}{S_{2}}\cdot \frac{\rho_{2}}{\rho_{1}}\right)\gamma }.$$

## 4. Conclusions

In conclusion, we have described the diffuse reflectance spectroscopy with dilution—a method being able to independently obtain relative absorption and scattering spectra of strongly absorbing materials. The use of this method is especially promising for halide perovskites, since these materials have strong absorption and a complex energy structure of transitions. This method allows to measure powders and pulverized single crystals, which significantly expands applicability of the method compared to transmission spectroscopy of thin films. We have demonstrated the applicability of this method to obtain reliable data on the energy structure of 3D perovskites and their low-dimensional analogues, including doped ones. The method allows to distinguish exciton and interband absorption, as well as defect bands and tail states. We hope that the addition to the existing and extremely widespread DRS experimental technique discussed in our work will open up for many groups the possibility of obtaining reliable and physically significant results concerning the optical structure of halide perovskites and their low-dimensional analogues.

## Data Availability

Not applicable.

## Sample Availability

Samples of the compounds are not available from the authors.

## Abbreviations

The following abbreviations are used in this manuscript:

DRS	Diffuse reflectance spectroscopy
FWHM	Full width at half maximum
